# Sex‐specific role of integrins in influencing tauopathy onset in htau mice

**DOI:** 10.1002/alz.088995

**Published:** 2025-01-03

**Authors:** Katie Bretland, Li Lin, Sarah J Terrill, Christine M. Dengler‐Crish

**Affiliations:** ^1^ Northeast Ohio Medical University, Rootstown, OH USA; ^2^ Carthage College, Kenosha, WI USA

## Abstract

**Background:**

Integrins are essential mediators of numerous critical cellular processes. Increasing evidence indicates that aberrant function of αVβ1 integrins contributes to the onset and progression of tauopathy. Previously, our group showed that the neuroprotective exercise hormone irisin—a known αVβ integrin modulator, prevented age‐related increases in phosphorylated tau and inflammation in hippocampus of presymptomatic‐age female but not male htau tauopathy‐model mice (Bretland et al., 2021). Here, we investigated whether presymptomatic‐age female and male htau mice exhibited intrinsic differences in hippocampal αVβ integrins and whether irisin treatment impacted integrin expression and downstream signaling related to tauopathy disease mechanisms (i.e. PI3K/Akt/GSKβ pathway).

**Methods:**

Presymptomatic age (4‐month old) htau mice were administered 1 month of irisin (100 ug/kg i.p.) or vehicle control injections. Age‐ and sex‐ matched C57BL/6J mice were used as controls. An additional cohort of presymptomatic age ovariectomized and surgical sham female htau and C57BL/6J mice also underwent 1 month of irisin/vehicle treatment. Mice were sacrificed at 5 months of age, hippocampi were fresh dissected, and automated capillary‐based protein quantitation via Protein Simple Wes/Jess platform was used to measure hippocampal levels of αVβ1 integrins, relevant pathway targets, and neuropathology.

**Results:**

Presymptomatic female htau mice had endogenously dysregulated β1 integrin expression compared to female controls; no such differences were found among males. Irisin treatment significantly reduced hippocampal αV integrin expression and trended towards decreasing β1 integrin expression in female mice while having no impact on integrin expression in males. Irisin treatment also significantly reduced intrinsically upregulated PI3K/Akt/GSKβ pathway signaling in female htau mice, but had no impact on this signaling in males. Ovariectomy prevented irisin’s neuroprotective effects against hippocampal ptau and inflammation in female htau mice; notably, irisin triggered an upregulation of β1 integrin expression in hippocampus of ovariectomized female htau mice.

**Conclusions:**

Alterations in αVβ integrin function may influence early tauopathy disease mechanisms in a sex‐specific manner. These findings emphasize a need to understand the role of integrins in dementia and emphasize the importance of identifying sex‐specific disease etiologies.

